# Towards an Understanding of Circatidal Clocks

**DOI:** 10.3389/fphys.2022.830107

**Published:** 2022-02-25

**Authors:** Alberto Rock, David Wilcockson, Kim S. Last

**Affiliations:** ^1^Department of Science, Scottish Association for Marine Science, Oban, United Kingdom; ^2^Institute of Biological, Environmental, and Rural Sciences, Aberystwyth University, Aberystwyth, United Kingdom

**Keywords:** circadian, circatidal, lunar, chronobiology, marine, intertidal, mechanistic understanding, evolution

## Abstract

Circadian clocks are an intrinsic element of life that orchestrate appropriately timed daily physiological and behavioural rhythms entrained to the solar cycle, thereby conferring increased fitness. However, it is thought that the first archaic ‘proto-clocks’ evolved in ancient cyanobacteria in a marine environment, where the dominant time cues (zeitgebers) probably would have been lunar-driven and included tidal cycles. To date, non-circadian ‘marine clocks’ have been described with circatidal (~12.4 h), circasemilunar (~14.8 days), and circalunar (~29.5 days) periodicity, mostly studied in accessible but temporally complex intertidal habitats. In contrast to the well-described circadian clock, their molecular machinery is poorly understood, and fundamental mechanisms remain unclear. We propose that a multi-species approach is the most apposite strategy to resolve the divergence that arose from non-circadian clockwork forged in an evolutionary environment with multiple zeitgebers. We review circatidal clock models with a focus on intertidal organisms, for which robust behavioural, physiological, or genetic underpinnings have been explicated, and discuss their relative experimental merits. Developing a comprehensive mechanistic understanding of circatidal clocks should be a priority because it will ultimately contribute to a more holistic understanding of the origins and evolution of chronobiology itself.

## Introduction and Background

For most of us, chronobiology is the study of the circadian clock in terrestrial species. However, life evolved in our oceans, harbouring a tremendous diversity of organisms and some of the most dynamic and extreme ecosystems on Earth. This has led to dramatic zonation of species and habitats (Dudgeon et al., 1999; Hofmann, 2001) which are highly temporo-spatially variable. Links between chronobiology and ecology are apparent but often underexplored regarding temporal plasticity and the concept of ‘chronotype’, which define the characteristic temporal properties of organisms and are the targets of selection (Helm et al., 2017). Warming oceans, especially at high latitudes, are driving habitat range expansion (Poloczanska et al., 2016) and exposure to different seasonal patterns of entrainment (and thermal/photoperiodic mismatches), limiting entrainment (Schmal et al., 2020) or setting up potential barriers of migrations (Huffeldt, 2020). Investigating marine clocks using the right approaches and tools (Mat, 2019) provides an understanding of clock flexibility/lability and ultimately may be relevant to even our own behaviour and behavioural disorders, such as sleep and mood, when considering the interplay of different, or disrupted, rhythms in single organisms (Häfker and Tessmar-Raible, 2020). This mini-review will focus on circatidal clocks most studied in intertidal animals, their origins, their zeitgebers, diversity, and future research directions. We do not describe the molecular cogs of circadian clocks for which there are many excellent reviews (e.g., Zheng and Sehgal, 2012). Similarly, circalunar clocks have been comprehensively reviewed elsewhere (Andreatta and Tessmar-Raible, 2020) and will only be fleetingly covered.

### What Is the Evolutionary History of Circatidal Clocks?

To understand the clocks of marine organisms, we must appreciate the origins of the circadian clock. There is a line of evidence, based on the evolutionary genomics of the *kaiA*, *kaiB*, and *kaiC* gene cluster in blue green algae, that suggests our ancient ancestors, the cyanobacteria, may have utilised a proto-clock as long as 2.5 billion (Ga) years ago (Tauber et al., 2004). The marine unicellular cyanobacterium *Prochlorococcus* is the most abundant and ancient photosynthetic organism on Earth, with ancestral lineages found in the fossil record ~3.2 Ga (Heubeck, 2009) that were adapted to low-oxygen, low-light, and high-nutrient conditions. *Prochlorococcus* evolved chlorophyll machinery and retained phycobilisomes, protein assemblages capable of absorbing light efficiently in generating energy and oxygen (Ulloa et al., 2021). Together with their freshwater relatives *Synechococcus*, *Prochlorococcus* is not only credited for oxygenating the planet ~2.3 Ga but was also the first to evolve a functioning biological clock (Golden et al., 1997). In both species, the clock consists of proteins KaiA, KaiB, and KaiC, with 24 h oscillations of KaiC phosphorylation persisting *in vitro*. It is suggested that possessing this endogenous timer may have been crucial in anticipating environmental extremes with active motile avoidance at a time when the atmosphere permitted significant UV radiation at the water’s surface (Pittendrigh, 1993; Hoiczyk, 2000).

By the time the first Eukaryotes emerged a billion years later, the stage was set for the rise of the modern trans-nuclear circadian clock. Putative early multicellular organisms, such as *Grypania*, probably either a bacterial colony or an alga (Han and Runnegar, 1992), may have been the first to evolve a clock, now mirrored in contemporary marine algae such as *Acetabularia*. Furthermore, the temporal environment in which the proto-clock evolved would have been very different to the present day. During the time of the first Eukaryotes 2.7–1.6 Ga, the rotational period of the earth is estimated to have been between 13.5 and 4 h (Krasinsky, 2002)! Given that the current circadian period is 24 h, the molecular clock must have had the flexibility to adapt to a changing zeitgeber and opens the question: how may this proto-clock have evolved and diversified through these geological timescales? We can say with some certainty (but little evidence) that since the first proto-clocks evolved in an aquatic environment, we should examine the marine environment for clues to the early evolution of chronobiology, including the deep-sea, considered analogous to regions of the primitive ocean (Mat et al., 2020), and investigate whether circatidal clocks are the precursors to the circadian clock (Wilcockson and Zhang, 2008). To do so, we should first consider the cyclic complexity of the marine realm.

### Cyclic Complexity of Intertidal Environments

**Figure 1:**
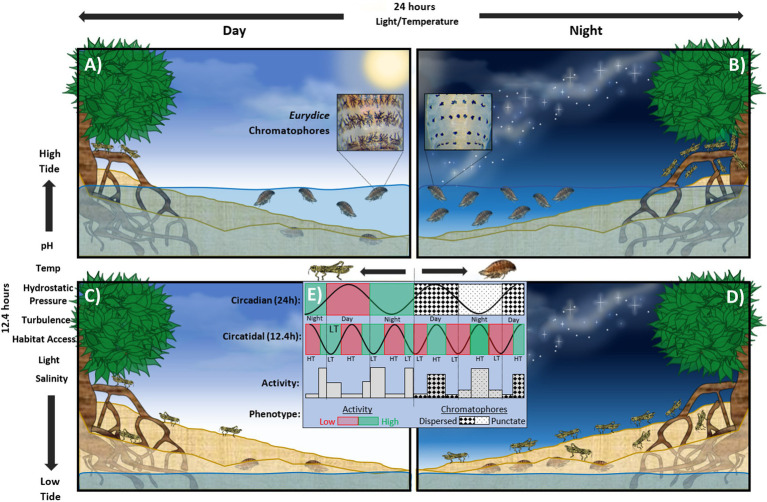
Representation of clocks of the speckled sea louse, *Eurydice pulchra* and the mangrove cricket *Apteronemobius asahinai* on a hypothetical shore. **(A)** Diurnal high tide; *Eurydice* appears more pigmented (circadian behaviour) and is active in the water column (circatidal behaviour). *Apteronemobius* is active (circadian) but does not forage (circatidal). **(B)** Nocturnal high tide; *Eurydice* appears less pigmented but is very active in the water column. *Apteronemobius* is not active. **(C)** Diurnal low tide; *Eurydice* appears pigmented but is buried in the sand. *Apteronemobius* is actively foraging on the shore. **(D)** Nocturnal low tide; *Eurydice* appears less pigmented and is buried. *Apteronemobius* is active but does not forage. **(E)** Summary of phenotypes as they relate to the circadian and circatidal cycles. Principle environmental zeitgebers for circatidal and circadian rhythms shown to the left and above panels, respectively.

The intertidal zone is dominated by the relentless ebb and flow of the tides, which vary in magnitude and range depending on seasonality, atmospheric conditions, and geography. Abiotic cycles linked to tidal movements include salinity, pH, temperature, turbidity, nutrient availability, and hydrostatic pressure, as well as diel, lunar, and seasonal events which superimpose on these dramatic changes (de la Iglesia and Johnson, 2013), each carrying ecological implications (Figure 1). These cycles are driven by gravitational and centrifugal forces of the Earth and moon spinning around a common centre of gravity and modulated by the gravitational effects of the sun over the annual cycle. The lunar day (the time it takes the earth to complete one revolution beneath the moon) takes 24.8 h and results in a single diurnal tide, i.e., as found in the Caribbean Sea or, more commonly, two high and low tides a day known as semidiurnal tides, with a period of ~12.4 h. When the sun and moon align (full and new moon), their combined gravity generates larger ‘spring’ tides, and when at 90^°^ relative to the earth (quarter moons), generate smaller ‘neap’ tides. These are known as semilunar tides, with a period of ~14.8 days, repeated every ~29.5 days but under a full moon (i.e., light at night) as the lunar cycle.

### What Is the Evidence for Circatidal Clocks?

Circatidal (12.4 h) rhythms were first observed in the intertidal acoel worm *Symsagittifera* (formerly *Convoluta*) *roscoffensis* by Gamble and Keeble (1903). After collecting the animals from the shore into laboratory tanks the researchers noted: ‘After a spell of insolation, colonies sink below the surface [of the sand], and after a certain sojourn in darkness they return to the surface. These movements synchronise with the covering and uncovering of the *Convoluta* zone by the tides’. The worms’ innate ability to anticipate the tidal cycle enabled predictive downward migrations into the sand even when removed from the shore. Later, several studies revealed similar behaviour under constant conditions in a number of other marine organisms including molluscs (Brown et al., 1954; Morton, 1977) and crustaceans (Enright, 1963; Morgan, 1965; Barnwell, 1966; Jones and Naylor, 1970). Naylor (1958) conducted a seminal study which demonstrated circatidal behaviour in the crab *Carcinus maenas* but modulated by a circadian ‘suppressor’ of activity. This revealed that such rhythms were generated endogenously without external stimulus in the laboratory. In the following years, more work contributed to the diversity of marine taxa displaying biological rhythms (see Naylor, 2010). Other classic examples of circatidal behaviours include locomotory and metabolic activity in the isopod *Eurydice pulchra* (Hastings, 1981a; Wilcockson and Zhang, 2008; O’Neill et al., 2015) and foraging behaviour in the mangrove cricket, *Apteronemobius asahinai* (Satoh et al., 2008; see Figure 1).

### How Are Circatidal Clocks Entrained?

The greatest difference between marine and terrestrial (predominantly circadian) clocks is in the mode and mechanism of entrainment. The marine component of circasemilunar rhythmicity in *Clunio marinus*, for example, is entrained by tidal cycles of temperature and mechanical disturbance (Neumann, 1978). Conversely in the congener *Clunio tsushimensis*, circasemilunar rhythmicity is entrained by moonlight and is suspected to be driven by an endogenous circasemilunar oscillator (Neumann, 1988, 2014). That the circasemilunar mechanisms in two species of midge from the same genus are entrained by different stimuli supports the putative diversity of marine clocks; if not in the fundamental clockwork, then at least the transducer linking environmental stimuli to the endogenous oscillator. Circatidal swimming in *E. pulchra* conversely (Figure 1) is likely entrained by a combination of synchronised wave action or vibration (turbulence) and variation in hydrostatic pressure as associated with benthic dwellers covered by different depths of water over a tidal cycle (Jones and Naylor, 1970; Hastings, 1981b; Zhang et al., 2013). Common tidal zeitgebers include pH, temperature, salinity and hydrostatic pressure (Reid and Naylor, 1990), vibration (Zhang et al., 2013), air/water exposure (Williams and Naylor, 1969), turbulence (Klapow, 1972; Neumann and Heimbach, 1984; Reid and Naylor, 1986), habitat access and immersion cycles (Chabot et al., 2008), and food pulses (Williams and Pilditch, 1997), which all vary cyclically with the tides (Figure 1).

### How Have Circatidal Rhythms Been Explained?

At a mechanistic level, there are several hypotheses which aim to address this question from different angles (Kim et al., 2003). Enright (1976) first proposed the bimodal clock hypothesis in which a single oscillator commands both circatidal and circadian rhythms, depending on the dominant zeitgeber (Enright, 1976). Such a clock could be fundamentally circadian, but entrainable by tidal zeitgebers such as water current. Indeed, it has been shown that in the oyster, *Crassostrea gigas*, tidal rhythms can be entrained by 6 h of light and dark cycles with circadian rhythms entrained by water current cycles (Mat et al., 2012, 2014).

While the bimodal clock hypothesis is simple in that it abrogates the need for dual endogenous oscillators, Palmer (1995) and Naylor (1996) each proposed mechanisms to reconcile circadian and circatidal rhythms. Palmer (1995) proposed the Circalunidian Clock Hypothesis, while Naylor (1996) the Circatidal/Circadian-Clock Hypothesis. Palmer tendered that in the fiddler crab *Uca pugnax*, circatidal behaviours are the effect of dual circalunidian clocks with a period of ~24.8 h coupled in antiphase, while Naylor argued that these behaviours were better explained by the interaction of a ~24 h circadian clock and a distinct ~12.4 h circatidal clock, at least in the green crab *Carcinus maenas*. While both hypotheses have their merits, there has been mounting evidence supporting the latter including the independent disruption of the circadian rhythm, while leaving circatidal behaviour unaffected in both *E. pulchra* and *A. asahinai* (Takekata et al., 2012, 2014a,b; Zhang et al., 2013).

Separate circadian and circatidal clockwork, as proposed by Naylor, also provide an explanation for seemingly endogenous semilunar rhythms, given their natural harmonic relationship; the Beat Hypothesis (Bünning and Müller, 1961) states that independent circadian and circatidal clocks would come into phase every ~14.8 days thereby generating a circasemilunar rhythm of behaviour coincident with the spring/neap tidal cycle (Neumann, 2014; Kaiser and Neumann, 2021). Tidal-related rhythms may also emerge where sensitivity to an exogenous stimulus is regulated by the circadian clock, explained by the Coincidence Detection Hypothesis (Kaiser and Neumann, 2021). Tidal turbulence is strongest during the rising high tide, which occurs at the same time of day every ~14.8 days. Therefore, if the circadian clock was to regulate the sensitivity of turbulence receptors to engage at a consistent time of day, the turbulence of the rising tide would be detected once every ~14.8 days and produce a circasemilunar stimulus (Kaiser and Neumann, 2021).

## Discussion

The nature of circatidal clocks has been extrapolated from overt behavioural rhythms in many marine organisms over the last century (see Naylor, 2010). The value of behavioural studies in describing rhythmic phenotypes controlled by the circatidal clockwork is undeniable, but a definitive mechanistic model of the core tidal oscillator and entrainment pathways is needed. To establish a testable mechanistic model of the circatidal clockwork, contemporary *functional* molecular and cellular approaches must be used against the rich backdrop of rhythmic tidal phenotypes, and across multiple species.

### How Do We Decipher the Molecular Basis of Circatidal Clocks?

Genomic and transcriptomic datasets on circadian ‘clock genes’ in rhythmic marine species have provided insights to the relationships between species and the temporal expression profiles of whole transcriptomes, yet functional analyses are lacking (with a few exceptions, see below), and with few attempts at deciphering how tidal (and semilunar and lunar) clocks actually work. For example, functional and loss of function strategies applied to marine species include *in vivo* genome/transcription manipulation and mutagenesis—RNA interference (Takekata et al., 2012, 2014a; Zhang et al., 2013; Payton et al., 2017), transcriptional activator-like effector nucleases (TALENs; Bannister et al., 2014), and rhythmic phenotype rescue in transgenic flies expressing clock, or clock-associated genes from marine animals (Beckwith et al., 2011; Zhang et al., 2013). Pharmacological perturbation of clock biochemistry (such as casein kinase inhibition), clock cell localisation (*in situ* hybridisation and immunochemical detection) and *in vitro* cell-based protein interaction assays (such as fly S2 cell systems) have been used to shed light on the role of canonical circadian genes in tidal and lunar rhythmicity (Zantke et al., 2013; Zhang et al., 2013). The paucity of functional studies on circatidal clocks likely reflects that organisms exhibiting robust, persistent and observable phenotypes lack tried and tested, species-specific and validated laboratory reagents or, in many cases, genomes. Many marine species also exhibit complex lifecycles, usually with planktonic phases, that make genome editing a significant challenge.

Nevertheless, it is increasingly evident that non-model systems are essential in revealing the nature and evolution of clocks and so called ‘wild clocks’ have emerged as a fertile ground for research (Schwartz et al., 2017). The breadth of marine species explored for their rhythmicity represents an exciting platform for targeted mechanistic approaches to gain insight to circatidal clock function. To facilitate this, good models should have some key attributes: a reliable and measurable circatidal phenotype (due to the inherent variability in tidal cues and to decipher between daily and truly tidal rhythms); genetic and experimental tractability; availability, accessibility, and simple husbandry.

Several suitable candidate non-model organisms have been identified. For example, *E. pulchra* has been shown to exhibit robust, entrainable phenotypes and is relatively content in experimental settings. However, its protracted development means that genome editing is challenging. *Apteronemobius asahinai* also shows promise as a tidal model (Satoh and Terai, 2019) and has a recently published draft genome (Satoh et al., 2021). Other models include an intertidal limpet (Schnytzer et al., 2018) and bivalves (Connor and Gracey, 2012; Mat et al., 2012, 2014, 2016; Gracey and Connor, 2016; Payton et al., 2017) but for the limpet in particular, individual, entrainable phenotypes are not as easily measured as for more mobile animals that lend themselves to infra-red beam monitoring. Therefore, energies and resources across multiple emerging model species are necessary; each model has its merits, and it is a tantalising prospect that concerted and collaborative efforts might yield deep insight into the evolution of the circatidal clockwork.

Circadian genes have been the focus of tidal and lunar clock research until now and there is evidence that dominant cycles of the organism (Mat et al., 2016, 2020; Tran et al., 2020), or even the demands of photosynthetic symbionts (in the case of the coral, *Aiptasia diaphana*), shape tidal or daily gene expression (Sorek et al., 2018). Such studies are intriguing but do not directly address the functional basis of circatidal phenotypes. In parallel with the manipulation of circatidal genomes to dissect the tidal clock (Takekata et al., 2012, 2014a; Zhang et al., 2013; Payton et al., 2017), efforts might also focus on identifying regulatory elements of tidally rhythmic gene transcription (tidally responsive DNA enhancers; TyDEs; O’Neill et al., 2015). These are suggested for nuclear expression of mitochondrial transcription factors in *E. pulchra* and were revealed by examination of metabolic markers of tidal activity. The recent discovery of a cell-autonomous 12 h murine clock regulated by the spliced form of X-box Binding Protein 1 (XBP1s) is interesting because XBP1s-driven rhythms of transcription are distinct and independent from the circadian clock. This feature resonates with the notion of independent circadian and tidal clocks. Moreover, XBP1s are conserved through evolution and have been described in some marine species (Pan et al., 2020; Tong et al., 2021); analysis of 12 h cycling transcripts in two intertidal species, the anemone *Aiptasia diaphana* and limpet *Cellana rota*, revealed significant overlap with 12 h cycling transcripts in mice (Pan et al., 2020).

### Where Is the Circatidal Clockwork?

The hypothesis that some tidal (and indeed, lunar) clocks are distinct from the circadian clock (Reid and Naylor, 1989; Takekata et al., 2012, 2014a,b; Zantke et al., 2013; Zhang et al., 2013; Satoh, 2017) raises questions about the physical interactions of clock cells with different periods and whether discrete subsets of neurons interact to drive circatidal phenotypes. There have been remarkably few attempts to describe the neuroarchitecture of clocks in lunar and tidal organisms with efforts hampered by the current lack of *bona-fide* tidal candidate genes or proteins to target. Some reagents, including antisera to circadian proteins, have been generated specifically for circatidal marine species such as *E. pulchra* (Zhang et al., 2013), while heterologous sera have been used to describe the brain regions expressing circadian neuropeptides or proteins in the mangrove cricket (Takekata et al., 2014b) and crabs (Beckwith et al., 2011). Attempts at isolating the cellular foci of tidal clocks using optic lobe ablation in the mangrove cricket have been reported and corroborate the notion of separate tidal and daily clocks (Takekata et al., 2014b). Efforts to generate and collaboratively share resources for cell localisation and functionality would be well rewarded.

### Understanding Circatidal Entrainment Mechanisms

The diversity of zeitgebers in coastal habitats offers the opportunity to explore how these environmental signals are transduced into the oscillator(s) of tidal organisms, a relatively untouched area of marine chronobiology; indeed, environmental perception *per se* in marine animals is poorly researched. For example, *E. pulchra* entrain to cycles of vibration or mechanical stimulation and *Drosophila* chordotonal organs, peripheral mechano-receptors, have been linked to the circadian entrainment *via* periodic vibration (Simoni et al., 2014) and temperature (Sehadova et al., 2009). A recent, elegant study on *Platynereis dumerilii* segmentally iterated r-opsin expressing peripheral sensory cells demonstrated dual photo and non-photosensory (mechanosensory) roles. Here, mechanosensory roles of the r-opsin receptors may have evolved secondarily to light receptors (Revilla-I-Domingo et al., 2021). Exploration of entrainment pathways from receptor to oscillator in other evolutionarily ancient marine species might yield important comparative outcomes with relevance to extant circadian systems.

## Conclusion

The first molecular proto-clocks probably evolved in the same organisms which paved the way for all aerobic life by oxygenating Earth’s atmosphere. These adapted to an ever-changing cyclic environment over geological timescales, including lengthening of the diel cycle. In the last century, many marine behavioural rhythms have been identified and multiple mechanisms suggested. Now, the rapid advance of powerful cellular and molecular methods has equipped chronobiologists with the necessary tools to reveal the clockwork in non-model marine organisms. Only through collaborative and interdisciplinary research linking marine biologists, molecular and cellular biologists, and neuroscientists, will we achieve a holistic understanding of the evolution, mechanism, and principles of the circatidal clock.

## Author Contributions

All authors listed have made a substantial, direct, and intellectual contribution to the work, and approved it for publication.

## Funding

This work was supported by the CHASE project, part of the Changing Arctic Ocean (CAO) programme, jointly funded by the UKRI Natural Environment Research Council (NERC, project number: NE/R012733/1) and the German Federal Ministry of Education and Research (BMBF, project number: 03F0803A). Additional funding was provided by the CAO project Arctic PRIZE (NE/P006302/1).

## Conflict of Interest

The authors declare that the research was conducted in the absence of any commercial or financial relationships that could be construed as a potential conflict of interest.

## Publisher’s Note

All claims expressed in this article are solely those of the authors and do not necessarily represent those of their affiliated organizations, or those of the publisher, the editors and the reviewers. Any product that may be evaluated in this article, or claim that may be made by its manufacturer, is not guaranteed or endorsed by the publisher.
